# Monomeric Banana Lectin at Acidic pH Overrules Conformational Stability of Its Native Dimeric Form

**DOI:** 10.1371/journal.pone.0062428

**Published:** 2013-04-26

**Authors:** Javed M. Khan, Atiyatul Qadeer, Ejaz Ahmad, Raghib Ashraf, Bharat Bhushan, Sumit K. Chaturvedi, Gulam Rabbani, Rizwan H. Khan

**Affiliations:** Interdisciplinary Biotechnology Unit, Aligarh Muslim University, Aligarh, India; University of Hyderabad, India

## Abstract

Banana lectin (BL) is a homodimeric protein categorized among jacalin-related family of lectins. The effect of acidic pH was examined on conformational stability of BL by using circular dichroism, intrinsic fluorescence, 1-anilino-8-napthalene sulfonate (ANS) binding, size exclusion chromatography (SEC) and dynamic light scattering (DLS). During acid denaturation of BL, the monomerization of native dimeric protein was found at pH 2.0. The elution profile from SEC showed two different peaks (59.65 ml & 87.98 ml) at pH 2.0 while single peak (61.45 ml) at pH 7.4. The hydrodynamic radii (*R*_h_) of native BL was 2.9 nm while at pH 2.0 two species were found with *R*_h_ of 1.7 and 3.7 nm. Furthermore at, pH 2.0 the secondary structures of BL remained unaltered while tertiary structure was significantly disrupted with the exposure of hydrophobic clusters confirming the existence of molten globule like state. The unfolding of BL with different subunit status was further evaluated by urea and temperature mediated denaturation to check their stability. As inferred from high C_m_ and ΔG values, the monomeric form of BL offers more resistance towards chemical denaturation than the native dimeric form. Besides, dimeric BL exhibited a Tm of 77°C while no loss in secondary structures was observed in monomers even up to 95°C. To the best of our knowledge, this is the first report on monomeric subunit of lectins showing more stability against denaturants than its native dimeric state.

## Introduction

Understanding the factors that decide how a linear polypeptide chain folds into its unique three-dimensional structure relic one of the fundamental questions in biology. Several models for the mechanism of protein folding have been proposed and there is ample argument concerning competing hypothesis. Current sources of content consist of whether there are on-pathway intermediates in the folding and if so, what the nature of such partially folded intermediates is. There are substantial evidences which indicate that proteins of smaller molecular weight under appropriate conditions fled to the native state within a few milliseconds with no detectable intermediate [1], [2]. Such folding is consistent with smooth-funnel energy landscape models. On the other hand, for many proteins there are extensive experimental data for transient intermediate, including the molten globule [3], [4]. The molten globule is an intermediate state of protein stabilized by acidic/alkaline pH, moderate concentration of strong chemical denaturants at a certain temperature. The general properties of this intermediate state are the presence of marked secondary structures, massive or significant loss of tertiary structures together with exposed hydrophobic clusters [5]–[7]. The available literatures support that the molten globule state (and other nonnative states of protein molecule) can stay in living cells and can be mixed up in a number of physiological processes owing to the fact that the negative electrostatic potential of the cell surface may attract protons from the immensity of solution leading to a local drop in pH, thus resulting in partial denaturation of proteins [8], [9]. There are now abundant data available regarding the molten globule state of monomeric protein, which has greatly improved our knowledge about protein folding problem and folding intermediates [10]–[12].

**Figure 1:**
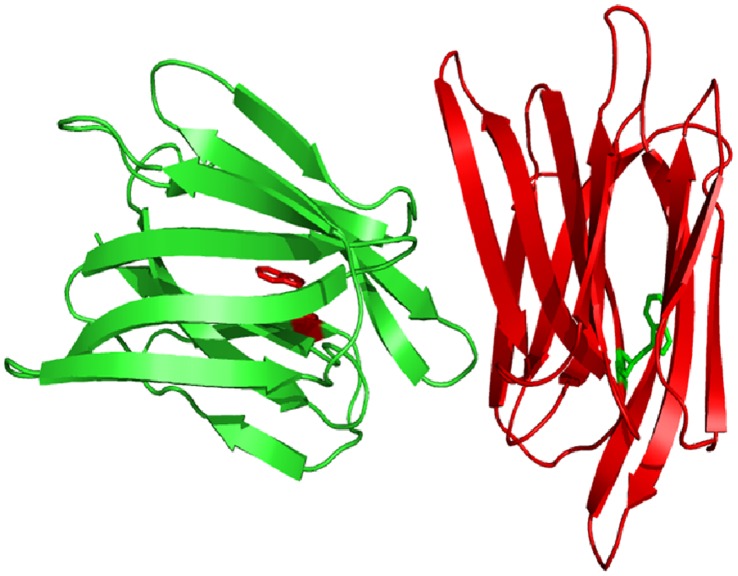
Ribbon structure of a banana lectin (2BMY) generated from PyMol. Trp residues are shown in both subunits.

Lectins are (glyco) proteins which specifically bind to mono or oligosaccharide reversibly. Lectins are found in every kingdom of life. They perform various biological functions ranging from host-pathogenic interaction, cancer metastasis, cell–cell communication, embryogenesis, mitogenic stimulation as well as the tissue development [13]–[16]. Their high affinity and specificity for glycoconjugates have found many applications in biological and biomedical research [17]. Banana lectin (BL) is a member of jacalin-related superfaimly of lectins and explicitly binds to mannose and glucose containing oligosaccharides [18], [19]. It is a homodimeric protein where each subunit has a molecular weight of 14.5 kDa and consists of 141 amino acid residues with a single Trp at position 10 (Figure 1). Also each subunit has twelve β-strands arranged in a β-prism-I fold. BL is known to act as a potent inhibitor of HIV replication which is main perpetrator in the list of fatal diseases [20]. In this work, we investigated the unfolding of BL at acidic pH using several spectroscopic as well as hydrodynamic techniques. The stability of the protein was compared under different conditions using temperature and chemical denaturants. In several instances, the individual subunits of dimeric/oligomeric proteins are reported to be less stable than native and biologically functional state of protein as it is believed that interchain interactions bind the subunits and impart additional stability to the dimer/oligomer. However to best of our knowledge, this is the first report on the monomeric state of protein being more stable than its native dimeric state.

## Materials and Methods

### Materials

Bananas were purchased from local shops. Urea, 1-analino-8-naphthalene sulfonate (ANS) Sephadex-75 were purchased from Sigma Chemicals Co. USA. All other chemicals used were of analytical grade.

### Protein Purification

BL was purified by the procedure of Koshte et. al. [21]. 250 g of banana pulp was soaked in 1 L distilled water containing 250 mM NaCl, 5 mM MgCl_2_ and 5 mM CaCl_2._ The mixture was subjected to homogenization followed by drop wise addition of 4 M NaOH to maintain pH upto 7.4. The homogenate was incubated for 15 min, 0.1 M glucose was added to the final volume and stirred for 2.0 h at room temperature. The solution was centrifuged at 18000 rpm for 30 min. The pellet was discarded, 85% ammonium sulfate was added to the supernatant and kept overnight for precipitation followed by centrifugation at 18000 RPM for 45 min. The pellet was collected and redissolved in Tris-HCl buffer of pH 7.4 and dialyzed for overnight. The clear suspension was loaded onto Sephadex-G75 affinity column. The specific elution was carried out using 0.5 M glucose in buffer (pH 7.4). The eluent was dialyzed to remove the glucose and lyophilized for further use. The purity of lectin was checked by SDS-PAGE and the concentration was determined by Lowry and BCA methods.

### Buffer Preparation

pH measurements were carried out on Mettler Toledo pH meter (Seven Easy S20–K) using Expert “Pro3 in 1” type electrode having the least count of 0.01 pH unit. The acid denaturation of BL was carried out in 20 mM of following buffers: Tris-HCl (pH 7.0–7.4), sodium phosphate (pH 6.0), sodium acetate (pH 3.5–5.0), glycine-HCl (pH 1.6–3.0) and KCl-HCl (pH 0.8–1.4). All the buffers were filtered through 0.45 µm syringe filter. Before all of the spectrophotometric measurements the protein samples were incubated for overnight at room temperature.

### Circular Dichroism

CD measurements were performed with a Jasco spectropolarimeter (J-815), equipped with a Jasco Peltier-type temperature controller (PTC–424S/15). The instrument was calibrated with D-10-camphorsulfonic acid. The measurements were carried out at 25°C. Spectra were collected with a protein concentration of 0.15 mg ml^−1^ with 0.1 cm path length and 1 mg ml^−1^ with 1 cm path length for far-UV and near-UV CD respectively. Each spectrum was the average of 2 scans. The results were expressed as mean residual ellipticity (MRE) in deg cm^2^ dmol^−1^ which is defined as:
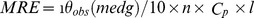
(1)where θ_obs_ is the CD in millidegree, n is the number of amino acid residues in one subunit (141 for BL), l is the path length of the cell in centimeters and Cp is the molar fraction of proteins.

### Thermal and Chemical Denaturations

The thermal unfolding of BL was carried out by heating the samples and measuring the temperature-dependent CD response at 218 nm from 25°C to 95°C using a temperature rise of 1°C /min in a water-jacketed cell attached to the Multitech M-H-03 water circulator. For chemical denaturation experiments, protein samples (0.15 mg ml^−1^) were allowed to equilibrate overnight with 0–10 M urea at desired pH.

### Fluorescence Measurements

Fluorescence measurements were performed on Hitachi spectrofluorometer (F-4500). For acid and chemical denaturation, the fluorescence spectra were measured with a protein concentration of 0.15 mg ml^−1^. The intrinsic spectra were recorded between 300 to 400 nm with excitation wavelength of 295 nm. For light scattering measurements, the excitation and emission wavelength was set at 350 nm as used in our previous report [22]. Both the excitation and emission slit widths were set at 5 nm. For extrinsic fluorescence measurement, the protein samples were incubated with 50-fold molar excess of ANS at room temperature in dark. The samples were excited at 380 nm and emission spectra were recorded between 400–600 nm.

### Acrylamide Quenching Experiments

For Acryl quenching experiments, aliquots of 5 M acrylamide (quencher) stock solution were added to the protein samples (0.15 mg ml^−1^) to achieve the desired range of quencher concentration (0–0.12 M). Excitation wavelength was set at 295 nm in order to excite Trp residues only and the emission spectrum was recorded in the range 300 to 400 nm. The decrease in fluorescence intensity at λ_max_ was analyzed according to the Stern–Volmer equation:
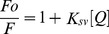
(2)where F_o_ and F are the fluorescence intensities at an appropriate wavelength in the absence and presence of quencher respectively, *K*_sv_ is the Stern–Volmer constant and [Q] is the concentration of the quencher.

### Dynamic Light Scattering (DLS)

DLS measurements were performed with protein concentration of 1.0 mg ml^−1^ using DynaPro–TC–04 dynamic light scattering equipment (Protein Solutions, Wyatt Technology, Santa Barbara, CA) equipped with temperature-controlled microsampler. Before the measurements, all the samples were kept for overnight (12 hrs) incubation. Prior to scanning, all the solutions were spun at 10,000 rpm for 15 mins and filtered through microfilter (Millipore Millex-HV hydrophilic PVDF) having a pore size of 0.45 µm followed by further filtration using 0.22 µm pore sized filter (Whatman International, Maidstone, UK). Measured size was presented as the average value of 50.0 runs. Data were analyzed by using Dynamics 6.10.0.10 software at optimized resolution. The mean hydrodynamic radius (*R_h_*) and polydispersity (P_d_) were estimated on the basis of an autocorrelation analysis of scattered light intensity based on the translational diffusion coefficient, by Stokes–Einstein equation:
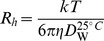
(3)where *R_h_* is the hydrodynamic radius, *k* is the Boltzman’s constant, T is the absolute temperature, *η* is the viscosity of water and 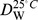 is the translational diffusion coefficient.

### Size Exclusion Chromatography (SEC)

Size exclusion chromatography of BL was performed at pH 7.4 and pH 2.0 on the 120 ml column from the Äkta Purifier (Amersham Bioscience AB, Uppsala, Sweden) using Sephadex G-75 matrix. The column was equilibrated with desired buffers containing 0.8 M Glucose and 0.15 N NaCl. The elution was carried out at a flow rate of 60 ml h^−1^ and the absorbance of eluted fractions was read at 280 nm. Blue dextran was used to determine the void volume of the column. Before running the SEC experiments the BL (1 mg ml^−1^) was incubated at pH 7.4 and 2.0 for overnight.

### Data Analysis

Chemical and thermal denaturation data from the CD and fluorescence spectroscopy were analyzed on the basis of two-state unfolding model. For a single step unfolding process, N U, where N is the native state and U is the unfolded state, the equilibrium constant *K_u_* is defined as
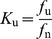
(4)With *f_u_* and *f_n_* being the molar fraction of U and N, respectivel.
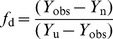
(5)Where Yobs, Yn and Yu represent the observed property, property of the native state and that of unfolded state respectively while 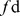
corresponds to the fraction of denatured protein. The change in free energy of unfolding in water 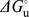 is obtained by the linear extrapolation method [23]. The relationship between the temperature and concentration of denaturant 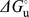 is approximated by the following equation:



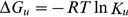
(6)and
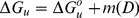
(7)where m is the experimental measure of the dependence of ΔG_u_ on temperature, R is the gas constant (1.987 cal K^−1^ mol^−1^) and T is 298 K. The concentration of denaturant at which the protein is half denatured (when ΔG_U_ = 0) is given by D_1/2_ and derived from equation 7 as follows:
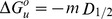
(8)


## Results

### Acid Induced Unfolding of BL

#### Far-UV CD measurements

**Figure 2:**
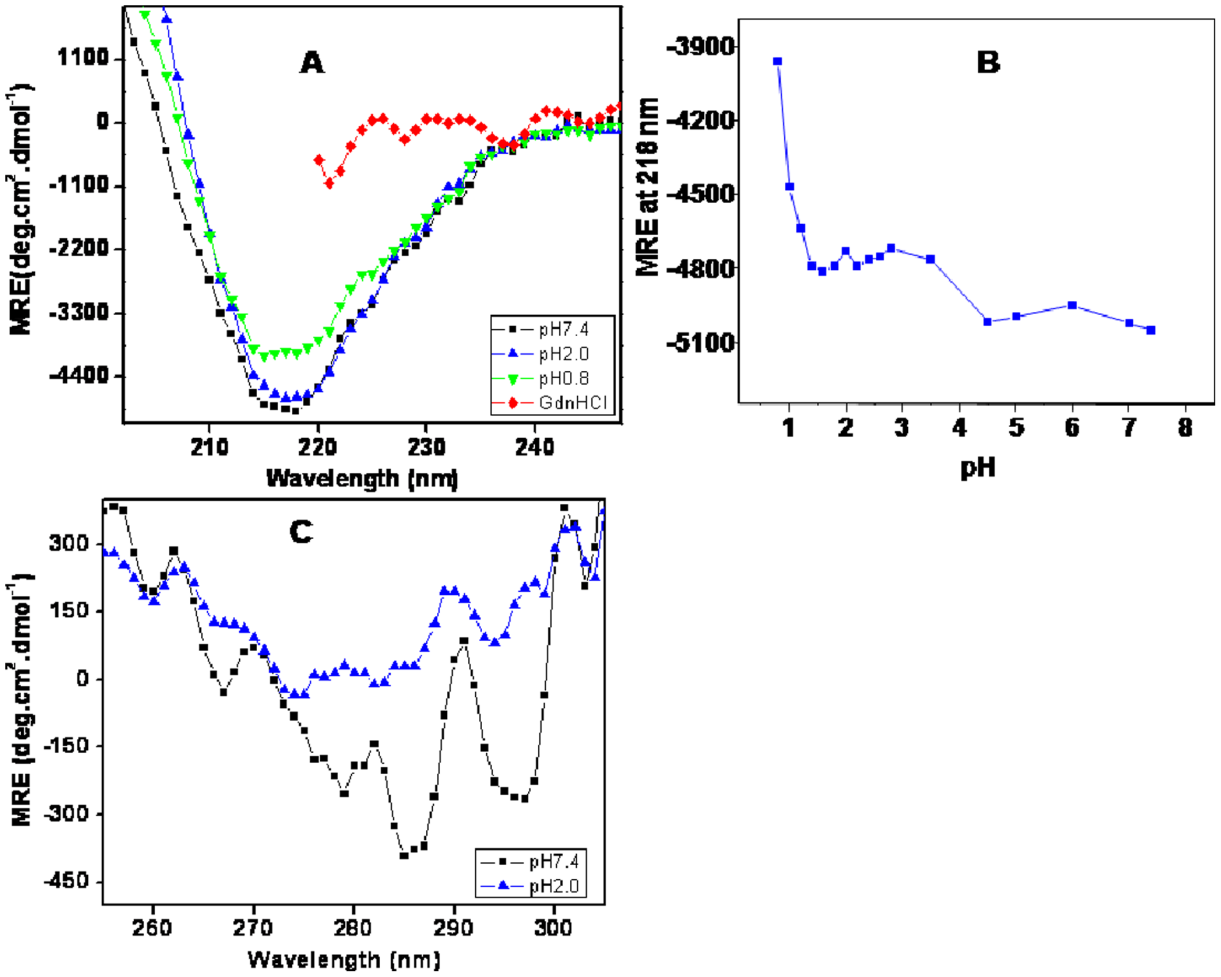
pH induced secondary structural change of BL. (A) Far-UV CD spectra of BL at pH 7.4 (-▪-), pH 2.0 (-▴-), pH 0.8 (-▾-) and 6 M GdnHCl (-♦-). (B) The change in mean residual elipticity (MRE) of BL at 218 nm was plotted with respect to pH. (C**)** Near-UV CD spectrum of BL at pH 7.4 (-▪-), and at pH 2.0 (-▴-). For far-UV CD and Near-UV CD measurements protein concentration was take 0.15 and 1.0 mg ml^−1^. BL was incubated at different pH overnight prior to measurements.

Far-UV CD is one of the most sensitive spectroscopic techniques for analyzing secondary structure of the proteins [24], [25]. A comparative change in far-UV CD spectra of BL at different pH is shown in (Figure 2A). Native state of BL (pH 7.4) exhibited a single negative peak at 218 nm giving an indication for the presence of β-conformation, a feature typical for several lectins [26]. The spectra of BL at pH 2.0 were quite similar to that of the native state (pH 7.4), a sign for considerable secondary structures being retained under acidic environment but in case of pH 0.8 a significant decrease in elipticity was found while the denatured state (6 M GdnHCl) appeared to have lost all the conformational elements. pH induced alteration in secondary structures of BL were further examined through change in MRE at 218 nm as shown in (Figure 2B). No change in ellipticity values was noticed in the pH range 1.4 to 7.4 indicating pronounced stability of BL against acidification. Further lowering of pH resulted in a noticeable decrease in ellipticity which suggested some loss of secondary structure driven possibly by the charge-charge repulsions occurring at extremely low pH.

### Near UV-CD Measurements

The CD spectra in near-UV (250 to 310 mm) range provides valuable information regarding changes in the environment of aromatic residues. The near-UV CD spectra of BL (Figure 2C) in native state revealed minima around 279 nm and 285 nm, a characteristic of buried aromatic chromophores particularly Trp [27]. The intensity of the signals was considerably diminished following incubation at pH 2.0. These data taken together with far-UV CD results suggested that BL retained significant secondary structure content with loose tertiary contacts at pH 2.0 and thus confirmed the existence of molten globule state at this pH.

### Intrinsic Fluorescence

**Figure 3:**
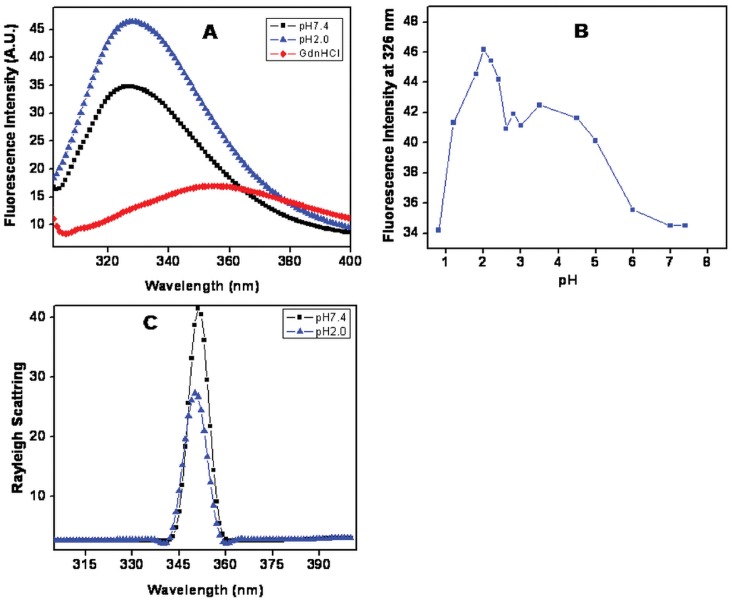
Effect of pH on the tertiary structure of BL was investigated by intrinsic fluorescence spectroscopy. (A) Intrinsic fluorescence spectra of BL at pH 7.4 (-▪-), pH 2.0 (-▴-) and 6 M GdnHCl (-♦-) after excitation at 295 nm. (B**)** Change in fluorescence intensity at 326 nm of BL was plotted against pH. (C) Rayleigh Scattering measurements of BL at pH 7.4 (-▪-) and pH 2.0 (-▴-) after excitation at 350 and spectra was measured in the range of 300–400 nm. All the measurements were performed after 12 hrs incubation in 20 mM of respective buffer and final protein concentration was taken 0.15 mg ml^−1^.

Intrinsic fluorescence is an important method to study protein conformation because it reveals the environment-dependent solvent exposure of the Trp indole ring and tyrosine aromatic side chains [28], [29]. The main intrinsic fluorescence probes of protein conformation, dynamics and intermolecular interactions are Trp, Tyr, and Phe. Out of these three, Trp is the most important probe because the indole ring is highly sensitive to its neighboring environment making it an ideal choice for reporting protein conformational changes and interactions with other molecules. The emission spectrum with excitation wavelength of 295 nm is mainly dominated by Trp fluorescence. As shown in (Figure 3A), native state of BL exhibited low fluorescence intensity (FI) and maximum fluorescence emission (λ_max_) at 326 nm indicating that the Trp residues are buried in the core of the protein. One possible explanation for the lower FI value at native pH is that the Trp fluorescence could be quenched by neighboring amino acid side chains such as Met76 of BL, which is in close proximity (∼4 Å, PDB: 2BMY) with the only Trp10 side chain [30], [31]. At pH 2.0, the emission maxima (λmax) of protein was red shifted by 3 nm along with significant increases in FI similar changes were also reported for diphtheria toxin [32]. For denatured state (6 M GdnHCl), λ_max_ was shifted to 353 nm indicating that Trp were maximally exposed to the solvent [33], [34]. The pH dependant changes in FI of BL at 326 nm are shown in (Figure 3B). A gradual increase in the FI was noticed following acidification up to pH 2.0 which together with significant red shift (from 326 to 329 nm) indicates that Trp residues were in a nonpolar environment [35], [36]. Due to the partial loosening of structure, Met76 and Trp10 residues might have displaced far apart to inhibit the quenching. The decrease in FI below pH 2.0 could be due to the intrinsic quenching of Trp fluorescence caused by conformational changes upon extreme acidification.

### Rayleigh Scattering Measurements

To check the possibility of pH dependent aggregation, we performed light scattering measurements. It is reported that if extent of light scattering is fivefold more in comparison to the native state so it is concluded that the solution is having aggregates [37]. The results are shown in (Figure 3C). As evident, the extent of light scattering at pH 2.0 was insignificant to that of native state and hence the chances of aggregation can be safely ruled out. As in our previous report we have found 12 times more scattering in the BL when it is incubated with SDS pH below two unit of pI [38].

### ANS Binding Studies

**Figure 4:**
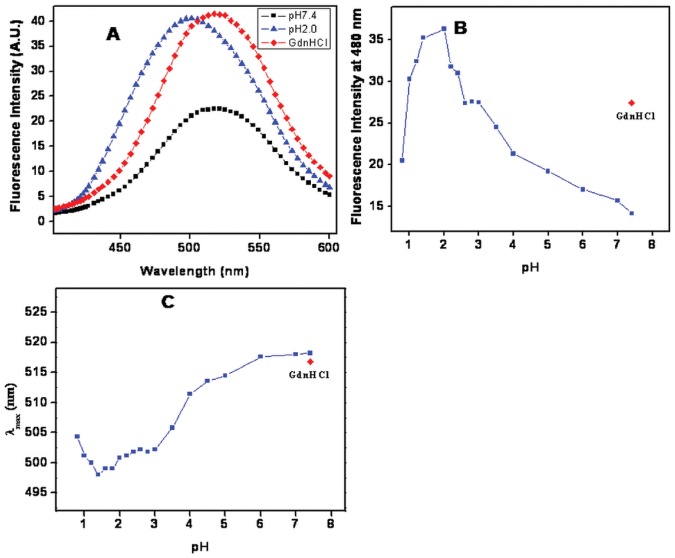
Change in Trp position was monitored by ANS dye binding. (**A**) Extrinsic fluorescence spectra of BL at pH 7.4 (-▪-), pH 2.0 (-▴-) and 6 M GdnHCl (-♦-). (B**)** pH dependant changes in ANS fluorescence intensity of BL at 480 nm. (C) Change in wavelength maximum of BL was plotted verses pH. Prior to measurements all the samples were incubated for 12 hrs in a 20 mM respective buffer with 0.15 mg ml^−1^ protein concentration and 50 times more ANS (265 µM) were added in all samples.

ANS is a widely used hydrophobic dye for detecting the non-native states such as molten globule (MG) states in proteins [39], [40]. Fluorescence spectra of BL with ANS at pH 7.4 and 2.0 (Figure 4A) revealed that binding of ANS to hydrophobic patches resulted in a prominent blue shift along with a significant increase in fluorescence intensity at pH 2.0 while at pH 7.4 the ANS is unable to bind BL because hydrophobic residues are buried in the core of the proteins. A plot of ANS fluorescence intensity at 480 nm as a function of pH is also shown in Figure 4B. ANS fluorescence was maximum at pH 2.0 suggesting considerable exposure of hydrophobic clusters that either remain inaccessible in native state (pH 7.4) or were minutely accessible in the denatured state of protein (6 M GdnHCl). Native state of BL exhibited maximum emission of ANS fluorescence at 519 nm which decreased to 501 nm at pH 2.0 (Figure 4C). Taken together, the above results confirm the existence of MG-state of BL at pH 2.0 having pronounced secondary structure along with significantly disrupted tertiary contacts and hydrophobic clusters considerably exposed to the solvent.

### Dynamic Light Scattering (DLS)

**Figure 5:**
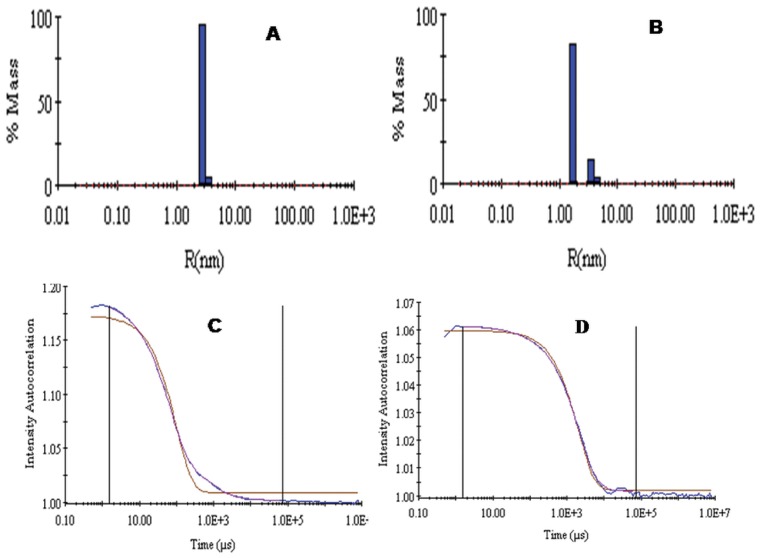
Change in hydrodynamic radii of BL at different pH were monitored by DLS. Hydrodynamic radii (*R*_h_) of BL at (A) pH7.4 and (B) pH 2.0 and autocorrelation graph of BL (C) at pH 7.4 and (D) at pH 2.0. BL (1 mg ml^−1^) were incubated at pH 7.4 and 2.0 for 12 hrs incubation.

The pH dependent changes in hydrodynamic radii (*R_h_*) were studied by DLS measurements. The *R_h_* of folded BL obtained at pH 7.4 was 2.9±1 nm (Figure 5A) with 7.8% polydispersity (Table 1), which is less than 30%, confirmed the homogeneity of molecules. At pH 2.0, *R_h_* values obtained were 1.7 nm and 3.7 nm pointing towards incomplete monomerization (Figure 5B). Besides, the increase in *R_h_* of the two species at pH 2.0 as compared to at pH 7.4 can be attributed to the perturbation of tertiary structure, thus supporting the existence of MG-state at this pH.

**Table 1:** Hydrodynamic radii (*R*_h_) of BL at different pH.

Conditions	*R_h_*(nm)	[%] PD
pH 7.4	2.9	7.8
pH 2.0	1.7,3.6	10.08, 12.05

### Size Exclusion Chromatography (SEC)

**Figure 6:**
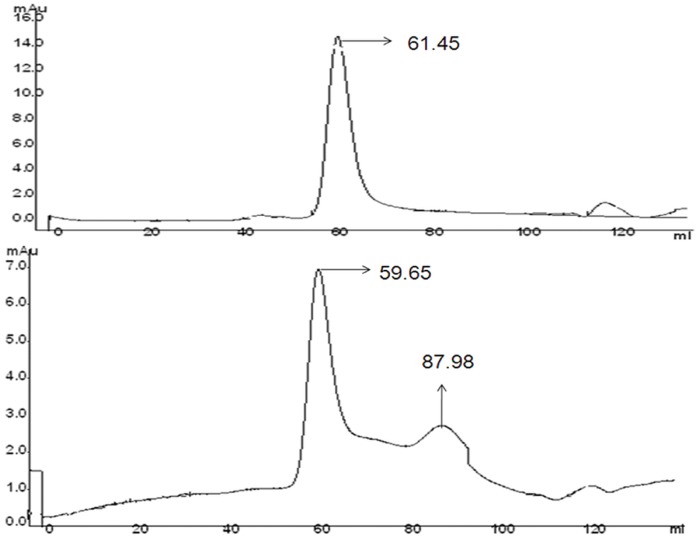
pH dependent monomerisation of BL. **S**ize exclusion profile of BL at pH 7.4 and pH 2.0. Before performing the experiments the BL (1 mg ml^−1^)was incubated at respective pH for overnight.

The elution profile of BL was obtained at pH 7.4 and pH 2.0 is depicted in (Figure 6) from size exclusion chromatography. A single peak around 61.45 ml was observed at pH 7.4 while the elution profile of protein at pH 2.0 revealed two peaks that were centered around 59.65 ml and 87.98 ml, although the later peak was not so sharp. The progressive monomerization of protein at pH 2.0 might have caused this peak to diminish resulting in only two peaks corresponding to the dimeric (59.65 ml) and relatively greater number of monomeric (87.98 ml) species. The distorted shape and lower elution volume of the later peak at pH 2.0 compared to at pH 7.4 can be attributed to the loss of tertiary structure [41]. The results establish that monomer of BL was found at pH 2.0 although complete monomerization was not found.

### Acrylamide Quenching Experiments

**Figure 7:**
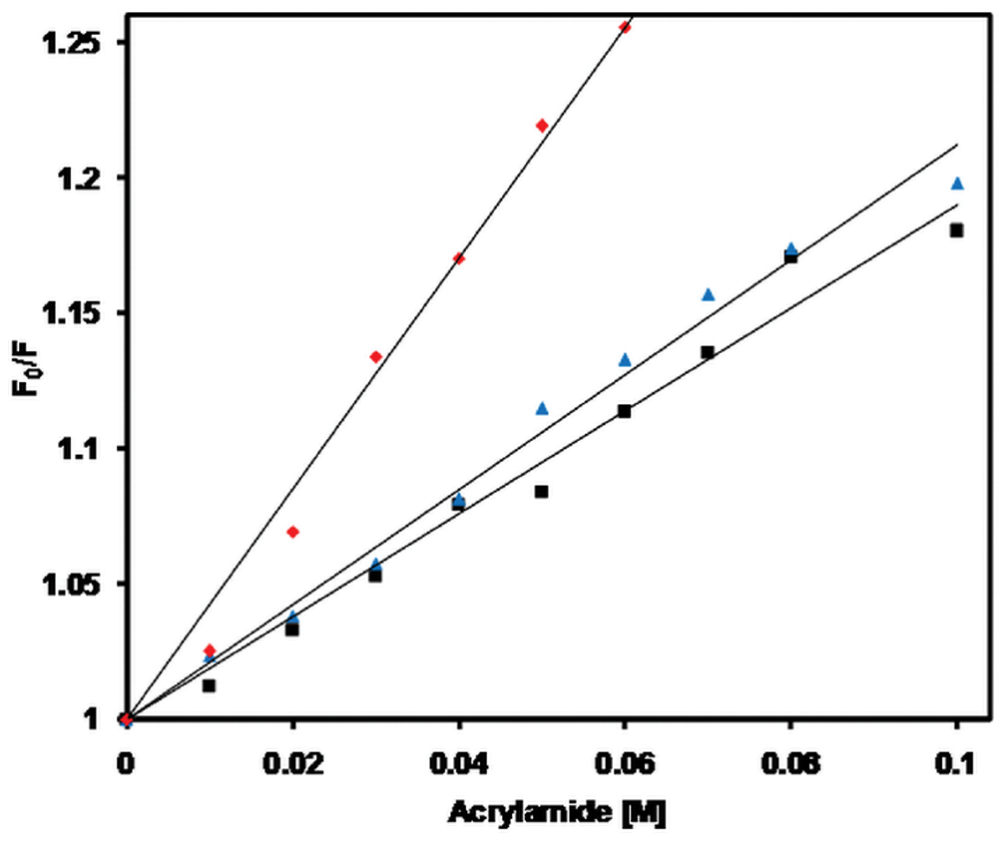
Exposure of Trp was monitored by acrylamide quenching. Stern–Volmer plot obtained from acrylamide quenching of BL at pH 7.4 (-▪-), pH 2.0 (–▴–) and in the presence of 6 M GdnHCl (–♦–).

To verify the environment of Trp residues, a fluorescence - quenching experiment was performed using the uncharged molecules of acrylamide as explained [42]. Figure 7 shows Stern–Volmer plot of BL at pH 7.4, 2.0 and in the presence of 6 M GdnHCl while Table 2 summarizes the *K*_sv_ obtained under respective conditions. The values for *K*_sv_ were in the order 4.48 M^−1^ (6 M GdnHCl) >2.01 M^−1^ (pH 2.0) >1.95 M^−1^ (pH 7.4). The data implied that Trp residues were maximally exposed in the presence of 6 M GdnHCl, relatively less exposed at pH 2.0, even lesser in case of pH 7.4 indicating that exposure of hydrophobic residues at pH 2.0 and in turn authenticated the destabilization of tertiary structure at pH 2.0 which validate the presence of a molten globule like state [43].

**Table 2:** Acrylamide quenching constant (*K*_sv_) values of native (pH 7.4), molten globule (pH 2.0) and denatured states (6 M GdnHCl) of BL obtained.

Conditions	*K*_sv_ M^−1^	R^2^
pH 7.4	1.95	0.98
pH 2.0	2.01	0.98
6 M GdnHCl	4.48	0.99

### Chemical and Thermal Denaturation of BL

The interesting results obtained from spectroscopic and hydrodynamic studies prompted us to compare the stability status of BL under native condition (pH 7.4) where protein existed as a homodimer and at molten globule state (pH 2.0) where monomeric BL predominated. The stability was checked using temperature unfolding in addition to urea as denaturants.

### Urea Induced Conformational Changes of BL at Native and Molten Globule State

Urea induced structural transitions of BL were monitored by far-UV CD and intrinsic fluorescence spectroscopic techniques.

### Secondary Structure Alterations

**Figure 8:**
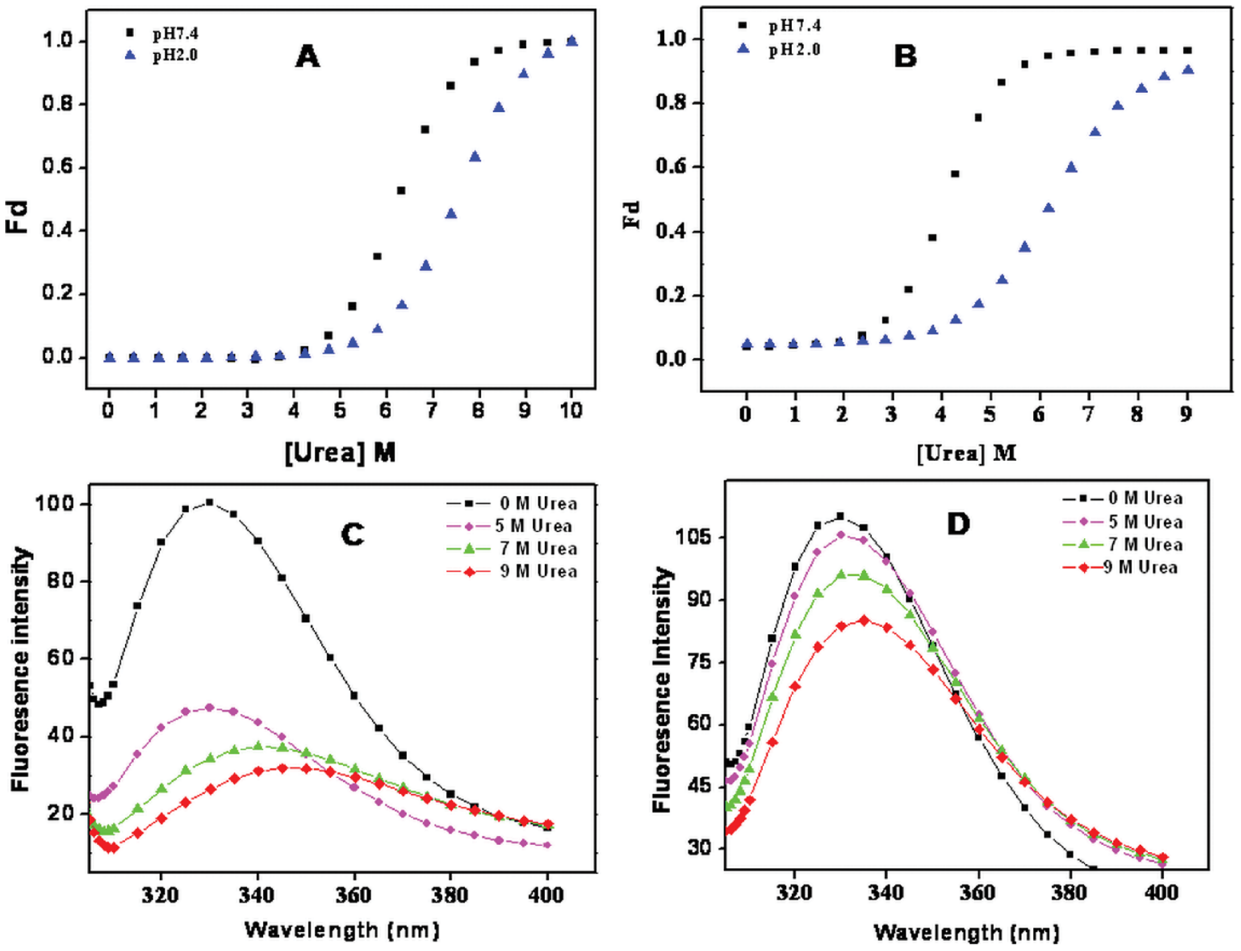
Urea-induced denaturation of BL was monitored by changes in secondary and tertiary structure. (A) Urea induced unfolding transition curves of BL at pH 7.4 (-▪-) and pH 2.0 (–▴–) monitored through ellipticity measurement at 218 nm by far-UV CD. (B) Urea induced unfolding of BL at pH7.4 (-▪-) and pH 2.0 (–▴–) monitored by a change in fluorescence emission intensity at 326 nm plotted as a function of urea concentration after excitation at 295 nm. (C) Fluorescence emission spectra of BL at pH 7.4 (8 C) and (D) at pH 2.0, at 0 M urea (-▪-), 5 M urea (-•-), 7 M urea (-▴-) and 9 M urea (-♦-).

Far-UV CD studies were carried out to study the effect of urea on the secondary structure of the BL at two different pH i.e. 7.4 and 2.0 respectively. Figure 8A, recapitulates the effect of increasing urea concentration on loss of ellipticity at 218 nm of BL. At pH 7.4, no significant change in ellipticity was observed up to 5 M. However, a large sigmoidal change was observed from 5 to 8 M urea. In case of pH 2.0, BL showed relatively more resistance and no secondary structure change was observed up to 6 M urea, followed by a large sigmoidal change that reached a plateau around 10 M Urea. The midpoint of sigmoidal denaturation (C_m_), an index to determine the stability of protein, was found to be 6.2 M for BL at pH 7.4. In case of pH 2.0, the C_m_ value increased to 7.4 M which indicated that the secondary structure of BL acquired more stability after minimization. The transition midpoint and free energy change (ΔG) of urea induced unfolding of BL at two pH values were determined by far-UV CD and fluorescence data are summarized in Table 3.

**Table 3:** Parameters characterizing the urea-induced unfolding of BL at different pH byusing fluorescence spectrofluorometer and Far-UV CD measurements.

Conditions		Methods	Transitionmidpoint (C_m_)	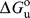 ^ (kcal/mol)^
pH 7.4, 25°C	Fluorescence spectrofluorometer	FI at 326 nm	4.1	3.13±0.20
	Far-UV CD measurements	MRE at 218 nm	6.2	3.85±0.085
pH 2.0, 25°C	Fluorescence spectrofluorometer	FI at 329 nm	6.1	3.43±0.088
	Far-UV CD measurements	MRE at 218 nm	7.4	5.44±0.133

### Tertiary Structure Alterations

The ureas induced tertiary conformational changes in BL at pH 7.4 and 2.0 and were also studied through changes in FI at 326 and 329 nm as a function of increasing concentration of urea. As shown in (Figure 8B), urea denaturation profile of BL at both conditions followed a two-state transition. At pH 7.4, structure of protein remained unaffected by 2 M urea while complete loss of structure was observed with 6 M urea resulting in a midpoint of transition at 4.1 M. At pH 2.0, the molecule unfolding initiated beyond 3 M urea and complete unfolding was achieved with 9 M of chaotrop. The C_m_ value shoot up to 6.1 M which clearly pointed towards a more stable state of BL being attained at pH 2.0 with respect to at native state. Similar to the native state of BL, the emission maximum in the absence of urea was 326 nm. The wavelength maximum of BL at pH 7.4 was started red shifting above 3 M urea concentration and maximum red shift were observed in the presence of 9 M urea with a difference of 22 nm. However at pH 2.0, the wavelength shift started at higher urea concentration but a shift in wavelength is not much more significant even at 9 M urea. The fluorescence spectra of BL at both pH 7.4 and 2.0 is shown in (Figure 8C and 8D). The above results clearly suggested that monomeric BL (pH 2.0) is chemically more stable than its dimeric form (pH 7.4).

### Thermal Denaturation

**Figure 9:**
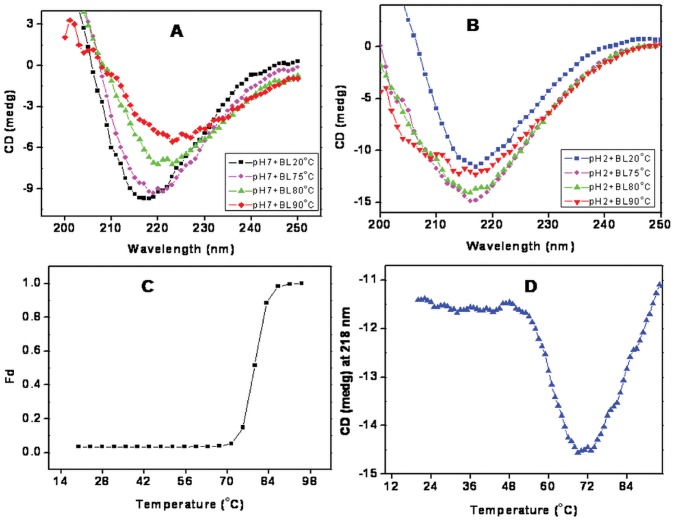
Effect of temperature on monomeric as well as dimeric form of BL. (A) temperature dependent change in far-UV CD spectra of BL at (A) pH 7.4, 20°C (-▪-) and (B) pH 2.0. 20°C (-▪-), 75°C (-•-), 80°C (-▴-) and 90°C (-♦-) (C) thermal denaturation profile of BL at pH 7.4 and (-▪-) (D) pH 2.0 (-▪-) obtained from changes in elipticity at 218 nm.

**Figure 10:**
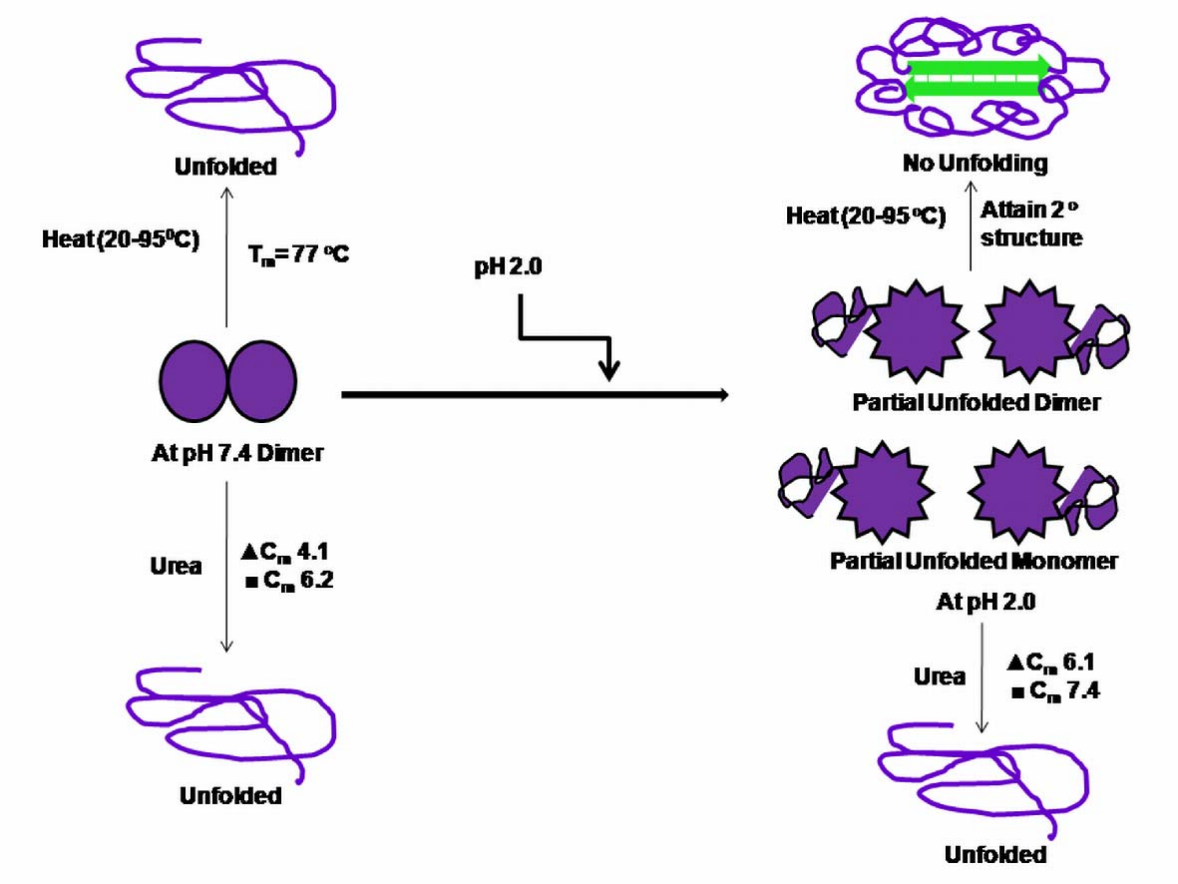
Schematic representation of the pH dependent conformational changes in BL as well as its urea and thermal induced unfolding. C_m_ values of BL obtained from fluorescence (▴) as well as far-UV CD (▪) measurements.

The chemical stability of BL was also complimented by thermal stability. Far-UV CD spectra of BL at pH 7.4 were taken at different temperatures (Figure 9A). No changes in spectra could be noticed up to 75°C beyond which a regular decrease in ellipticity was observed and spectra shifted towards higher wavelength due unfolding. Interestingly at pH 2.0 the spectra peak shifted towards shorter wavelength with increase in temperature (Figure 9B Besides, no significant loss of ellipticity was observed even at 95°C. Further, the temperature dependant changes in ellipticity at 218 nm of BL at were monitored and shown in (Figure 9C). The thermal denaturation profile of BL at pH 7.4 revealed sigmoidal transitions with midpoint of transition (*T_m_*) at 77°C. However at pH 2.0, no sigmoidal change was noticed over the entire temperature range (20–90°C) although a considerable increase in ellipticity was recorded up to 70°C suggesting induction rather than loss of secondary structure (Figure 9D). Thus, induction of secondary structure at pH 2.0 indicated uninterrupted gain of stability following monomerization. The results obtained from these experiments further strengthened the fact that acid-induced monomeric state, and not native dimeric form, of BL has more stability against temperature. Taken together, all these data further confirmed that BL acquires more chemical as well as thermal stability in its monomeric form than its native dimeric form. The overall results obtained in the present study are summarizes in (Figure 10).

## Discussion

Newly synthesized proteins are unfolded and do not have any structure. Over a short span of time, they attain various typical structures including secondary, tertiary and quaternary. The conformational elements acquired by polypeptides are stabilized by various non-covalent and covalent interactions. Breaking these interactions result in denaturation of the protein with either partial or complete loss of structure and its biological activity, that facilitates understanding of protein folding mechanism. The present study was divided into three parts: (i) pH denaturation (ii) chemical denaturation and (iii) thermal denaturation. The first part covers the pH dependent conformational change of BL to trace out any intermediate state that might encompass the folding pathway of protein. An intermediate state with characteristic features of a molten globule state was found to be populated at pH 2.0 as analyzed by CD, intrinsic as well extrinsic fluorescence methods. Size exclusion chromatography as well as DLS data confirmed that acid denaturation of BL was accompanied by monomerization of otherwise dimeric protein. A single peak corresponding to native BL was observed at pH 7.4. The signal intensity as well as elution volume was more for monomeric species of protein at pH 2.0. Besides, the *R_h_* value of both monomeric as well as dimeric species increased to 1.7 nm and 3.7 nm respectively owing to the conformation transition induced by extreme low pH as observed during CD and fluorescence analysis. The intramolecular charge-charge repulsion at extreme pH might have resulted in relatively extended conformation at low pH and similar explanation was also found in case beta-lactamase, cytochrome c, and apomyoglobin [44]. Besides, the polydispersity value indicated that the number of monomeric species were considerably high with respect to dimeric species at pH 2.0 suggested that acid-induced MG state of BL exist possibly in the former state. The pH dependant change in subunit status has been reported earlier for several multimeric proteins. The several noncovalent interactions that held the subunit of multimeric proteins are likely to be dissociated with change in pH [45]. The second part of this study was to compare the chemical stability of various states of BL. Chemical stability of BL determined at neutral pH using guanidine hydrochloride has already been reported showing that BL is quite stable [46]. In the present study, chemical stability of BL was examined at both monomeric (pH 2.0) as well as dimeric (pH 7.4) state using urea as a denaturant. Urea is widely exploited as chaotrops for checking the stability of proteins at neutral as well as acidic pH [47]–[49]. The equilibrium unfolding of BL in the presence of urea was found to be a cooperative process in which the protein molecule undergoes unfolding without stabilization of any partially unfolded intermediate at both pH values (7.4 and 2.0) similarly no intermediates were found in the case of GdnHCl denaturation of same lectin at pH 7.4 [46]. Oligomeric proteins have usually more chemical stability than its monomer form because of additional non-covalent and covalent interactions [50]. In case of BL, however, it was noticed that monomeric state (pH 2.0) of protein was more stable in comparison to dimeric native state which was evident from C_m_ values as well as free energy changes (Table 3). C_m_ values for BL at pH 7.4 as determined by far-UV CD and intrinsic fluorescence analysis were found to be 4.1 M and 6.2 M respectively. The difference in the two C_m_ value reflects the perturbation of two different conformational elements (secondary and tertiary structure). In case of monomeric BL, the C_m_ values increased to 6.1 M and 7.4 M respectively. Similarly, free energy change for unfolding of monomeric BL was more than dimeric protein which is quite an unusual finding this may be due to the development of positive charge on protein and exposure of hydrophobic patches, urea is probably not able to interact at its lower concentration because urea is a neutral molecule with negligible hydrophobic moiety. However, higher concentration of urea might have disturbed the overall charge on BL, consequently resulting in unfolding of the molecule. Previously it was reported that urea interact with proteins nonspecifically at sub-global level by hydrogen bonding and Van der Waals interaction with main chain and side chain groups of protein [51], [52]. Concanavalin A, Cramoll 1 and SBA are legume lectins which monomerize and form MG states up to 3 M urea [53]–[55] but this non-leguminous BL shows the tertiary structural changes from 4 M and secondary structural changes from 5 M urea which might be due to monomerization of native dimeric state of BL. This signifies that BL is more stable than other leguminous lectins. Urea concentrations up to 3–4 M affect BL in similar fashion at pH 2.0 or 7.4, after that the species formed on-pathway (possible monomers) are more stable in acidic conditions than at pH 7.4. Third part of this study was to check the stability of BL by employing temperature scan in the range of 20–95°C. Thermal denaturation of BL was monitored by far-UV CD spectroscopic techniques aimed to follow secondary structural changes of the protein. Thermal denaturation of BL was a cooperative process without any intermediate at pH 7.4 but an intermediate state was found at pH 2.0. T_m_ value of BL was quite high at pH 7.4 (77°C) indicating considerable stability against temperature change. Interestingly, the thermal denaturation profile was quite different at pH 2.0 where an intermediate state was acquired near 70°C which did not denature completely even when heated beyond 95°C, again confirming the significant increase in stability of BL with monomeric conditions.

### Conclusions

Acid denaturation of BL yielded an intermediate state at pH 2.0 and the monomerization of homodimeric BL was also found. Besides, the monomeric state of BL had more chemistry and thermal stability which, to the best of our knowledge, this is the first report on significant stability exhibited by an individual subunit of dimeric protein. An increase in intrasubunit interactions at monomeric level than in dimeric condition may be the possible reason for such finding.
